# Contemporary Databases in Real-world Studies Regarding the Diverse Health Care Systems of India, Thailand, and Taiwan: Protocol for a Scoping Review

**DOI:** 10.2196/43741

**Published:** 2022-12-13

**Authors:** Wen-Yi Shau, Sajita Setia, Salil Prakash Shinde, Handoko Santoso, Daniel Furtner

**Affiliations:** 1 Regional Medical Affairs Pfizer Corporation Hong Kong Limited Hong Kong Hong Kong; 2 Executive Office Transform Medical Communications Limited Wanganui New Zealand

**Keywords:** Asia, health care databases, real-world data, real-world evidence, scoping review

## Abstract

**Background:**

Real-world data (RWD) related to patient health status or health care delivery can be broadly defined as data collected outside of conventional clinical trials, including those from databases, treatment and disease registries, electronic medical records, insurance claims, and information directly contributed by health care professionals or patients. RWD are used to generate real-world evidence (RWE), which is increasingly relevant to policy makers in Asia, who use RWE to support decision-making in several areas, including public health policy, regulatory health technology assessment, and reimbursement; set priorities; or inform clinical practice.

**Objective:**

To support the achievement of the benefits of RWE in Asian health care strategies and policies, we sought to identify the linked contemporary databases used in real-world studies from three representative countries—India, Thailand, and Taiwan—and explore variations in results based on these countries’ economies and health care reimbursement systems by performing a systematic scoping review. Herein, we describe the protocol and preliminary findings of our scoping review.

**Methods:**

The PubMed search strategy covered 3 concepts. Concept 1 was designed to identify potential RWE and RWD studies by applying various Medical Subject Headings (MeSH) terms (“Treatment Outcome,” “Evidence-Based Medicine,” “Retrospective Studies,” and “Time Factors”) and related keywords (eg, “real-world,” “actual life,” and “actual practice”). Concept 2 introduced the three countries—India, Taiwan, and Thailand. Concept 3 focused on data types, using a combination of MeSH terms (“Electronic Health Records,” “Insurance, Health,” “Registries,” “Databases, Pharmaceutical,” and “Pharmaceutical Services”) and related keywords (eg, “electronic medical record,” “electronic healthcare record,” “EMR,” “EHR,” “administrative database,” and “registry”). These searches were conducted with filters for language (English) and publication date (publications in the last 5 years before the search). The retrieved articles will undergo 2 screening phases (phase 1: review of titles and abstracts; phase 2: review of full texts) to identify relevant and eligible articles for data extraction. The data to be extracted from eligible studies will include the characteristics of databases, the regions covered, and the patient populations.

**Results:**

The literature search was conducted on September 27, 2022. We retrieved 3,172,434, 1,094,125, and 672,794 articles for concepts 1, 2, and 3, respectively. After applying all 3 concepts and the language and publication date filters, 2277 articles were identified. These will be further screened to identify eligible studies. Based on phase 1 screening and our progress to date, approximately 44% (1003/2277) of articles have undergone phase 2 screening to judge their eligibility. Around 800 studies will be used for data extraction.

**Conclusions:**

Our research will be crucial for nurturing advancement in RWD generation within Asia by identifying linked clinical RWD databases and new avenues for public-private partnerships and multiple collaborations for expanding the scope and spectrum of high-quality, robust RWE generation in Asia.

**International Registered Report Identifier (IRRID):**

DERR1-10.2196/43741

## Introduction

### Background

Real-world data (RWD) related to patient health status or the delivery of health care can be broadly defined as data collected outside of conventional clinical trials. RWD are derived from a wide range of sources, including databases, treatment and disease registries, electronic medical records (EMRs), insurance claims, and information directly contributed by health care professionals or patients themselves [1].

High-quality, real-world evidence (RWE) relies on the appropriate analysis of RWD collected in ways that maximize their completeness, accuracy, standardization, and timeliness and reduce bias [2]. Yet, effective RWD utilization also requires disparate data sources to be turned into high-quality data sets [3].

Policy drivers have increased RWE adoption, particularly in the Western hemisphere where, for example, the US 21st Century Cures Act required the Food and Drug Administration to develop guidelines for the role of RWD in drug approvals [4]. In the United Kingdom, the Academy of Medical Sciences and the Association of the British Pharmaceutical Industry have recently prioritized supporting the inclusion of RWD in regulatory and health technology assessment processes, as well as the inclusion of electronic health records (EHRs), via the US Health Information Technology for Economic and Clinical Health Act and EHR incentive programs under the Affordable Care Act [3].

The collection of RWE is increasingly crucial in Asia. Only around 17% of clinical trials are conducted in Asia, and Asian populations are often underrepresented in pivotal clinical trials [5,6]. RWE provides certainty about the safety and effectiveness of medications, health interventions, and technologies in local settings for Asian patients [5]. Therefore, there is a need to increase the adoption of RWE by policy makers in Asia to support decision-making in several areas, including public health policy, regulatory health technology assessment, and reimbursement; set priorities; or inform clinical practice [7].

To support the purpose of achieving the benefits of RWE in Asian health care strategies and policies, we sought to identify the linked contemporary databases used in real-world studies from three representative countries—India, Thailand, and Taiwan—and reflect the diversity in Asia by performing a systematic scoping review.

The databases identified in our review will serve as a basis for further guiding approaches and initiatives that aim to drive collaboration and improvements in the generation and utilization of RWE in health care decision-making within Asia.

### Rationale for Selecting 3 Diverse Countries (India, Thailand, and Taiwan)

Asia is a very diverse region. For the planned scoping review, we chose a representative country for the following three economy types in Asia: high-income economy (Taiwan), upper-middle–income economy (Thailand), and lower-middle–income economy (India). These economies were defined according to the World Bank analysis for the 2023 fiscal year [8]; low-income economies are those with a gross national income (GNI) per capita (calculated using the World Bank Atlas method) of US $1085 or less in 2021, lower-middle–income economies are those with a GNI per capita of between US $1086 and US $4255; upper-middle–income economies are those with a GNI per capita of between US $4256 and US $13,205, and high-income economies are those with a GNI per capita of US $13,205 or more.

Another factor that contributed to our decision to focus on these three countries was the fact that market approval processes, including reimbursement, and price control mechanisms for medicines and medical devices are very distinct in Thailand, India, and Taiwan. India has a largely self-pay health care system through which patient payments are made to private sector providers [9]. Thailand and Taiwan provide health insurance for universal coverage. Thailand provides differential decentralized benefit packages to those who can contribute the premium, while a single social health insurance scheme exists in Taiwan. Additionally, Taiwan adopts more comprehensive payment system reforms, such as global budgeting, which contributes to cost containment [10]. Further, listing in the National Health Insurance formulary for Taiwan requires evidence of effectiveness, whereas cost-effectiveness is not mandatory in Thailand [11].

### Rationale for Our Study

Linked or integrated contemporary databases are like clinical data warehouses or repositories that could serve as excellent resources for the generation of RWD for disease surveillance, monitoring, and treatment outcomes and the timely detection of infection outbreaks [12,13]. However, little is known about the scope and competency of these databases, which are expected to vary based on the economies and health care reimbursement systems in different countries. Hence, the identification and thorough analyses of these databases are the first step in understanding their capabilities, trends, and variations in different countries within Asia and may enable future private-public research partnerships.

## Methods

The study protocol and methodology for our scoping review will adhere to the PRISMA-ScR (Preferred Reporting Items for Systematic Reviews and Meta-Analyses extension for Scoping Reviews) guidelines [14]. Herein, we discuss the approaches for the concept and research strategy, data extraction, and data mining.

### Concept Strategy and Filters

Articles will be retrieved by searching the National Institutes of Health’s PubMed database, using appropriate Medical Subject Headings (MeSH) terms and keywords together with appropriate filters, as described below. The following concepts were used to generate the search strategy for our scoping review.

#### Concept 1 (RWE and RWD Citations)

Since there are no MeSH terms for RWE and RWD, the MeSH terms and keywords in the following query were adapted from our own research and prior studies [15,16]: *(“Treatment Outcome”[MeSH] OR “Evidence-Based Medicine”[MeSH] OR “Retrospective Studies”[MeSH] OR “Time Factors”[MeSH] OR “real world” OR “real-world” OR “RWD” OR “RWE” OR “real life” OR “real patient” OR “real practice” OR “real clinical” OR “real population” OR “actual world” OR “actual life” OR “actual patient” OR “actual practice” OR “actual clinical” OR “actual population”)*.

#### Concept 2 (Pilot Countries):

The following query introduced the three countries: *(“India”[MeSH] OR “Taiwan”[MeSH] OR “Thailand”[MeSH] OR “India” OR “Taiwan” OR “Thailand”)*.

#### Concept 3 (Real-world Research Databases):

The MeSH terms and keywords in the following query were adapted from our own research and prior studies [16]: (“Electronic Health Records”[MeSH] OR “Insurance, Health”[MeSH] OR “Registries”[MeSH] OR “Databases, Pharmaceutical”[MeSH] OR “Pharmaceutical Services”[MeSH] OR “registry” OR “registries” OR “electronic health record*” OR “electronic healthcare record*” OR “electronic medical record*” OR “EHR” OR “EHRs” OR “EMR” OR “EMRs” OR “claims database*” OR “administrative database*” OR “hospital data” OR “claims data” OR “electronic health data” OR “clinical database*” OR “electronic healthcare data” OR “informatics”).

#### PubMed Filters

In addition to the three concepts outlined above, we also applied the following two filters in PubMed: a filter for English-language publications and a filter for publications within the last 5 years of the date of the search. We focused on English publications to help with identifying studies and RWD and RWE databases that would be of interest to an international audience. We also limited the search to articles published within the last 5 years to help with identifying databases that are currently being used or have recently been used.

### Research Strategy for Phases 1 and 2 of the Screening Process

Textbox 1 provides the inclusion and exclusion criteria for the extraction of data from the citations that were retrieved from PubMed by using the above search strategy. The eligibility criteria established for our scoping review were carefully chosen after the consideration of prior systematic reviews and scoping reviews and were selected to help with identifying studies reporting RWD and RWE. Of particular note, we will limit the studies to those involving data that were collected across more than 1 institution, similar to a previous report [17]. We made this decision because single-center databases may be less representative of a country or may include a highly specific patient population. We also decided to exclude pragmatic clinical trials (PCTs). Although such studies may fall within the scope of RWD, as reviewed in the *Discussion* section, these studies are subject to some limitations, and they are sometimes difficult to differentiate from randomized controlled trials (RCTs) [18,19], raising the complexity of the screening process.

A single reviewer performed phase 1 screening (titles and abstracts of publications) and phase 2 screening (full texts) to determine the eligibility of the publications for data extraction. Articles for which full texts were unavailable were also reassessed, and those that clearly satisfied the eligibility criteria were included in the next phase. Phase 1 and phase 2 screening were carried out by using Covidence software (Veritas Health Innovation Ltd), which is recommended by Cochrane. The software screens for duplicates automatically, although duplicates were considered unlikely due to the use of 1 database. The results will be further verified by a second reviewer. Any contradictions or discrepancies that arise between the reviewers will be discussed until a consensus is reached. If there is no consensus, a third reviewer will be consulted.

The hand searching of reviews, other publication types, or the reference lists of eligible articles is not planned. It was considered that any articles that would normally be identified via hand searching were more likely to predate the search filters or would be unlikely to satisfy the eligibility criteria.

**Inclusion and exclusion criteria for the extraction of data from the citations retrieved from PubMed.**

**Inclusion criteria**

- Database types
  Studies involving electronic health records, health insurance claims, administrative claims, clinical registries, or pharmacy databases
  Databases with research data involving >1 hospital or clinic
- Publication types
  Original research, including brief reports, short communications, and research letters
- Study types
  All types of real-world studies (or their protocols) using the following databases: electronic health records, health insurance claims, administrative claims, clinical registries, or pharmacy databases
- Scope of publication
  Studies with databases involving Taiwan, India, or Thailand
  Eligible international, regional, or multicountry studies will be included, provided that any of the target countries are included

**Exclusion criteria**

- Database types
  A data source involving electronic health records, health insurance claims, administrative claims, clinical registries, or pharmacy databases is not mentioned
  Databases with research data involving 1 hospital or clinic
- Publication types
  Correspondence and letters to the editor; editorials; commentaries; guidelines; case reports; case series (publications with prospective descriptions of a handful of patient cases; retrospective case series with a real-world data study design [20] will be eligible for inclusion); and narrative, systematic, or scoping reviews
- Study types
  Randomized controlled trials, pragmatic clinical trials, preclinical studies, and nonhuman studies
- Scope of publication
  Studies with a scope outside of Taiwan, India, or Thailand

### Data Extraction

After phase 2 screening, we plan to extract the following data from eligible studies:

1. Article characteristics, including the manuscript type (clinical study vs protocol), year of publication, and contact details of the corresponding author or database manager.
2. Database characteristics, including the region(s) covered, number of participating centers and institutions, and source of data (eg, medical records, health care insurance, clinical registries, pharmacy records, or mixed databases involving more than 1 type of data).
3. Study participant characteristics, including the number of subjects included in the primary analyses and the disease or medical condition studied.
4. Study types, including comparative effectiveness studies (involving clinical benefit, safety, quality of life or cost comparison of at least 2 treatments), single-population studies (eg, the burden of disease, epidemiology, disease nature course, or treatment pattern), and others. Studies will be further categorized based on the studied outcome(s).
5. Study duration (start and end year).

Eligible abstracts for which full texts are unavailable or cannot be sourced will be included for data extraction to maximize the availability of data from the largest number of published articles as much as possible. This was considered feasible because abstracts often contain the information that we wish to collect for data extraction.

A single reviewer will extract the data from all eligible full texts and abstracts by using a template on Covidence that was standardized based on the data extraction requirements. A second reviewer will review and perform a quality check of the extracted data.

### Data Mining

The key databases from each country will be analyzed based on the frequency of the use of each research database to generate published RWD studies that are written in English and indexed in PubMed. The number and characteristics of key databases from each country will be analyzed further for web-based research, and the frequency with which each major research database is used to generate published, English, PubMed-indexed RWD studies will be analyzed for each country.

## Results

We have finalized the search strategy, and Table 1 provides the final number of citations that were retrieved from PubMed via the search conducted on September 27, 2022. After applying the three concepts and the filters for language (English) and publication date (publications in the last 5 years before the search), the search yielded a total of 2277 citations. No duplicates were identified. Further citations may be identified during the screening and data extraction steps.

We have now started phase 1 screening (titles and abstracts) for the retrieved citations. Based on our progress to date, approximately 44% (1003/2277) of the articles were included in phase 2 screening. Of these, we anticipate that around 800 studies will be eligible for data extraction, based on the trend for the proportion of eligible studies in phase 2 screening (full texts) [17]. This accounts for approximately 35% (800/2277) of the articles retrieved via the literature search.

We hope to complete the pilot research and submit the results for publication in early 2023. Although our preliminary research involves 3 selected countries in Asia, we hope to expand our search to include studies from other countries based on the results of the scoping review for the three pilot countries.

**Table 1:** Final number of citations that were retrieved from PubMed via the search strategy.

Query number	Query	Results, n
1	Concept 1: *(“Treatment Outcome”[MeSH] OR “Evidence-Based Medicine”[MeSH] OR “Retrospective Studies”[MeSH] OR “Time Factors”[MeSH]* *OR “real world” OR “real-world” OR “RWD” OR “RWE” OR “real life” OR “real patient” OR “real practice” OR “real clinical” OR “real population” OR “actual world” OR “actual life” OR “actual patient” OR “actual practice” OR “actual clinical” OR “actual population”)*	3,172,434
2	Concept 2: *(“India”[MeSH] OR “Taiwan”[MeSH] OR “Thailand”[MeSH] OR “India” OR “Taiwan” OR “Thailand”)*	1,094,125
3	Concept 3: (“Electronic Health Records”[MeSH] OR “Insurance, Health”[MeSH] OR “Registries”[MeSH] OR “Databases, Pharmaceutical”[MeSH] OR “Pharmaceutical Services”[MeSH] OR “registry” OR “registries” OR “electronic health record*” OR “electronic healthcare record*” OR “electronic medical record*” OR “EHR” OR “EHRs” OR “EMR” OR “EMRs” OR “claims database*” OR “administrative database*” OR “hospital data” OR “claims data” OR “electronic health data” OR “clinical database*” OR “electronic healthcare data” OR “informatics”)	672,794
4	Query 1 *AND* query 2 *AND* query 3^a^	4168
5	Query 4 with filter for English-language articles	4163
6	Query 5 with filter for articles published in last 5 years^b^	2277

^a^(((“Electronic Health Records”[MeSH] OR “Insurance, Health”[MeSH] OR “Registries”[MeSH] OR “Databases, Pharmaceutical”[MeSH] OR “Pharmaceutical Services”[MeSH] OR “registry” OR “registries” OR “electronic health record*” OR “electronic healthcare record*” OR “electronic medical record*” OR “EHR” OR “EHRs” OR “EMR” OR “EMRs” OR “claims database*” OR “administrative database*” OR “hospital data” OR “claims data” OR “electronic health data” OR “clinical database*” OR “electronic healthcare data” OR “informatics”)) AND ((“India”[MeSH] OR “Taiwan”[MeSH] OR “Thailand”[MeSH] OR “India” OR “Taiwan” OR “Thailand”))) AND ((“Treatment Outcome”[MeSH Terms] OR “Evidence-Based Medicine”[MeSH] OR “Retrospective Studies”[MeSH] OR “Time Factors”[MeSH] OR “real world” OR “real-world” OR “RWD” OR “RWE” OR “real life” OR “real patient” OR “real practice” OR “real clinical” OR “real population” OR “actual world” OR “actual life” OR “actual patient” OR “actual practice” OR “actual clinical” OR “actual population”)).

^b^The search was conducted on September 27, 2022.

## Discussion

We aim to identify the medical and health-related databases used in 3 representative countries within Asia by applying a defined search strategy with a set of inclusion and exclusion criteria, as detailed in this protocol. By using this search strategy, we have already identified a large number of eligible studies from the three countries; our recent estimates suggest that around 35% (800/2277) of the studies that were retrieved via the literature search will be used for data extraction to identify and characterize the relevant databases used in India, Taiwan, and China. This rate is higher than that of a global review of articles published between 2010 and 2015, in which 10,069 articles were screened and 2635 unique data sources were identified (approximately 23%) [17]. This also indicates that our search strategy is focused and vigorous in identifying relevant citations for our research question.

We have excluded databases with research data involving a single hospital or clinic from the scope of our research to identify relevant linked clinical databases with the potential for adopting big data to generate robust, fit-for-purpose RWE in Asia. Our rationale for including databases with research data involving more than 1 hospital or clinic in the search strategy was to gather details on current databases that are linked across clinical centers, which enable holistic RWD generation with good external validity. A similar criterion was also applied in another recent study [17]. Single-center clinical data and study outcomes are known to have limitations, such as limited generalizability; small study effects; a higher risk of bias, including reporting bias; or limitations related to the selection of participants, treatment administration, and care providers’ expertise [21].

Asia is a highly diverse region, and our systematic scoping review will focus on 3 different countries that are deemed representative to reflect that diversity. We understand that the findings from the pilot research countries may not apply throughout Asia because the health care systems and database standards vary across the region. Hence, we hope to expand our research to include other countries in Asia after completing the preliminary research on the selected three countries. We are also mindful of the limitation that our findings will be limited to citations that are retrieved from PubMed only. Searching too few literature databases may yield a biased sample of primary studies, which may influence the accuracy of the summary effects and subsequently reduce the validity and generalizability of the systematic review results. Nevertheless, limiting the search to PubMed is in line with our strategy for identifying research databases that generate and yield robust RWD that are fully published in indexed, peer-reviewed journals and reducing duplicate search results.

We also excluded the PCT design, although it falls within the scope of RWD. PCTs are randomized studies in which the study participants should be similar to patients who would receive the intervention if it became usual care, which is information that may be unknown for new interventions. There are several limitations with the PCT design [18], and a recent review indicated that PCTs have a high degree of diversity in their designs and scopes, PCTs have deficiencies in reporting and trial registry data, and many studies with a pragmatic intent do not use the term *pragmatic* in the title or abstract [19]. Hence, including PCTs would have risked the methodology and complicated the retrieval of eligible studies. Therefore, we decided to exclude them in our search strategy. Likewise, RCTs were excluded because RWD are collected outside of highly controlled RCTs; thus, such studies would not serve the purpose of our review. The lack of randomization is a key criterion for identifying RWD studies and is well reflected in the definitions provided by reputable bodies, including the Association of the British Pharmaceutical Industry and the International Society for Pharmacoeconomics and Outcome Research [22]. Currently, the National Institutes of Health’s MeSH database lacks specific terms for RWE and RWD. Therefore, we relied on our own research and on strategies suggested in published studies, as explained in the *Methods* section, to identify relevant articles.

Overall, we believe that our research will be crucial for determining and understanding the scope, spectrum, and competence of linked, clinical, real-world databases in diverse countries with different economies and health care reimbursement systems. We anticipate that this crucial step will nurture advancement in RWD generation by shaping new avenues for public-private partnerships and multiple collaborations for high-quality, robust RWE generation in Asia.

## Abbreviations

EHR: electronic health record
EMR: electronic medical record
GNI: gross national income
MeSH: Medical Subject Headings
PCT: pragmatic clinical trial
PRISMA-ScR: Preferred Reporting Items for Systematic Reviews and Meta-Analyses extension for Scoping Reviews
RCT: randomized controlled trial
RWD: real-world data
RWE: real-world evidence

## Data Availability

All available data are included within this article.
